# Developing a sentinel syndromic surveillance system using school-absenteeism data, example monitoring absences over the 2020 COVID-19 pandemic

**DOI:** 10.1017/S0950268821002399

**Published:** 2021-11-09

**Authors:** Jennifer Lai, Helen E. Hughes, Roger Morbey, Paul Loveridge, Jamie Lopez Bernal, Vanessa Saliba, Esther Kissling, Alex Lovelock-Wren, Jeremy Mabbitt, Alex J. Elliot

**Affiliations:** 1 Public Health England, Real-time Syndromic Surveillance Team, Field Service, National Infection Service, Birmingham, UK; 2 Public Health England, National Disease Registration Service, Sheffield, UK; 3 Public Health England, Immunisation and Countermeasures Division, National Infection Service, London, UK; 4 Public Health England, COVID-19 Surveillance Cell, London, UK; 5 Epiconcept, Paris, France; 6 Studybugs, Brighton and Hove, UK

**Keywords:** Absence data, COVID-19, real-time surveillance, school-aged children, syndromic surveillance

## Abstract

This study describes the development of a pilot sentinel school absence syndromic surveillance system. Using data from a sample of schools in England the capability of this system to monitor the impact of disease on school absences in school-aged children is shown, using the coronavirus disease 2019 (COVID-19) pandemic period as an example. Data were obtained from an online app service used by schools and parents to report their children absent, including reasons/symptoms relating to absence. For 2019 and 2020, data were aggregated into daily counts of ‘total’ and ‘cough’ absence reports. There was a large increase in the number of absence reports in March 2020 compared to March 2019, corresponding to the first wave of the COVID-19 pandemic in England. Absence numbers then fell rapidly and remained low from late March 2020 until August 2020, while lockdown was in place in England. Compared to 2019, there was a large increase in the number of absence reports in September 2020 when schools re-opened in England, although the peak number of absences was smaller than in March 2020. This information can help provide context around the absence levels in schools associated with COVID-19. Also, the system has the potential for further development to monitor the impact of other conditions on school absence, e.g. gastrointestinal infections.

## Introduction

The coronavirus disease 2019 (COVID-19) pandemic progressed across the world in early 2020 and is presenting significant challenges to society [1]. The United Kingdom is currently (June 2021) progressing out of its third national lockdown. Infection rates and the severity of COVID-19 have predominantly affected the elderly, and those with underlying chronic health conditions [2]. Based on clinical and epidemiological data collected during the first pandemic wave, children accounted for a very small proportion of cases overall [3]. Although children are as likely to contract COVID-19 as adults and are thought to be important transmitters of infection [4], they are less likely to be symptomatic or develop severe symptoms [5]. One study found compared to adults, children aged 0–9 years were less likely to transmit the virus but older children aged 10–19 years were just as likely to spread the virus [6]. In England, following the first national lockdown children returned to school in September 2020 prompting concerns about an increase in education-based outbreaks leading to further increases in community infection in adults and vulnerable groups.

Syndromic surveillance is often used to supplement public health surveillance systems which track the progression of infectious diseases at a national, regional and local level [7]. Syndromic surveillance uses real-time non-specific symptom/preliminary diagnosis information, most often collected during routine healthcare provision, to show possible impact and provide an early indication of outbreaks of infectious diseases, non-communicable diseases and other health-related threats to a population [1]. Public Health England (PHE) currently maintain six national syndromic surveillance systems including NHS 111 online and telehealth phone calls, general practitioner in hour and out-of-hours consultations, emergency department attendances and ambulance calls. These systems monitor symptoms/conditions grouped as indicators, with surveillance outputs published in the public domain on a weekly basis. In 2020, these systems were adapted to respond to the COVID-19 pandemic with new syndromic indicators developed to monitor COVID-19-like symptoms at a national and regional level.

This paper aims to describe the development of a pilot sentinel syndromic surveillance system, using school absence data to monitor the impact of COVID-19 on school children in England as an example.

## Methods

Studybugs is a software platform for schools and includes an online free service (including an app) which is used by parents to report their children absent from school and prompts parents to report the reason for and symptoms related to that period of absence [8]. Based upon ‘reason for absence’, Studybugs is able to provide information to parents using symptom-specific PHE health protection recommendations [9]. Studybugs process the data and provide anonymised, open (freely available) data which are shared with schools and made available to other organisations with parental consent [8]. As open data, Studybugs provide an ‘application programming interface’ (API) for accessing anonymised data. Data are added as and when parents use the app to report an absence, with the majority (85%) of reports sent by 9 am each day. Data available from the Studybugs API include: anonymised report identifiers for each absence, anonymised subject identifiers, school information (school name and school town), date and time of report (this may not reflect the day the absence took place) and reason for absence (including common symptoms such as cough, runny nose or sore throat). In England, schools open Monday to Friday and are closed at weekends, with the academic year running from late August/September to July. Most schools have six holiday periods: October (1 week); December/January (2 weeks); February (1 week); March/April (2 weeks); May (1 week) and July/August (6 weeks).

Schools were eligible for inclusion here where they were located in England and provided data during both 2019 and 2020 calendar years. The daily ‘total’ number of absences (including all reports for any reason, not just sickness related) was calculated for each day during 2019 and 2020 calendar years. Additionally, the daily number of absences with ‘cough’ included as a reason for absence was calculated. Time series charts were constructed for both ‘total’ and ‘cough’ absences. School absence data exhibit large day of the week effects, with low activity at weekends and public holidays, therefore day-to-day fluctuations were smoothed by calculating 7-day moving averages for working days which accounted for public holidays and weekends [10, 11]. Only schools that participated in Studybugs during both 2019 (as a baseline year) and 2020 were included, to ensure that results were comparable across the study period. All analysis was undertaken using RStudio version 4.0.3 [12].

## Results

A total of 743 schools were included in the study as they participated in the app during both 2019 and 2020. A total of 378 332 absence reports were made for any symptom/condition/reason across the entire study period (186 334 in 2019, 191 998 in 2020). Of the ‘total’ absences, 59 117 included ‘cough’ as a reason for absence (28 076 in 2019, 31 041 in 2020). Both years were similar with around 63% of ‘total’ absence reports made for children from primary school, 35% from secondary school and 2% from other school types including nurseries and all-through schools (schools that include at least two stages of a child's education, often primary and secondary).

**Fig. 1.**
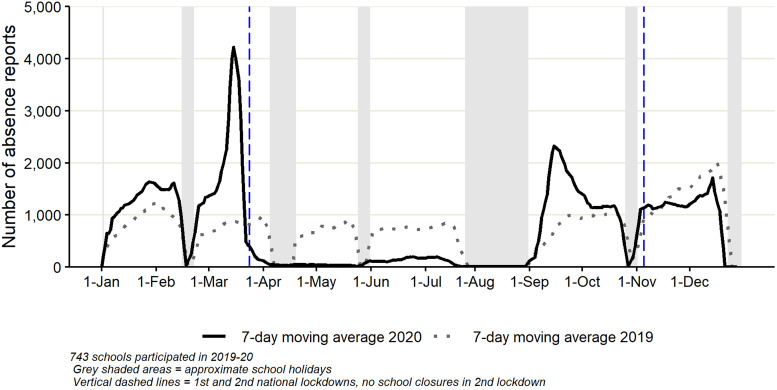
Daily number of ‘total’ school absence reports (presented as a 7-day moving average) reported to Studybugs during 2019 and 2020.

There were six troughs in the ‘total’ number of absence reports for 2019, which aligned with expected school holiday periods; in mid-February, April, late May, late July through to late August, late October and mid to late December. For 2019, overall, the number of absence reports decreased slightly between the Easter holiday (in April) and summer holidays (mid-July and August), and increased during the winter towards December (Fig. 1).

During 2020 there was a large spike in the ‘total’ number of absence reports in March, this was much higher compared to the same month in 2019 (Fig. 1). This was followed by a steep drop from the end of March 2020, with very low numbers of absences reported in April and May 2020. Numbers increased slightly in June 2020 although remained lower than 2019 levels, then decreased at the end of July 2020 and August 2020. The number of absence reports increased in September 2020, this increase was steeper and higher than the increase seen in September 2019 but not as high as numbers observed in March 2020. Numbers decreased from mid-September 2020. From October through to December 2020, the trend replicates the pattern observed for the same months in the previous year.

**Fig. 2.**
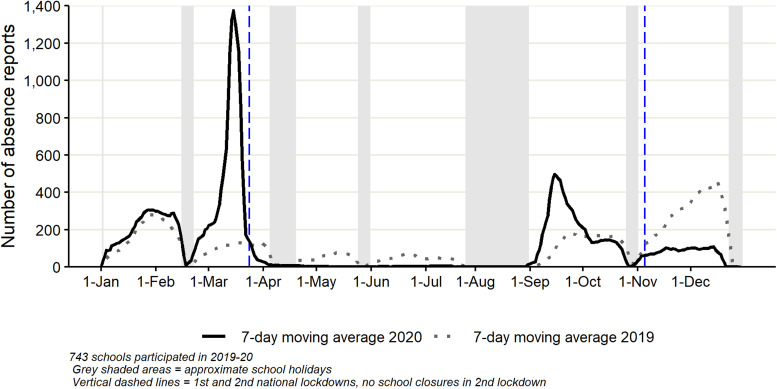
Daily number of ‘cough’ school absence reports (presented as a 7-day moving average) reported to Studybugs during 2019 and 2020.

Very similar numbers and trends in ‘cough’ absences were identified during January and February in both 2019 and 2020 (Fig. 2). Low numbers of ‘cough’ absences were reported in the early spring and summer period of 2019 (March to August), before an increase in September following return to school and another large increase during November/December, as seen in ‘total’ absences. The March 2020 increase in ‘cough’ absences was very rapid, followed by an equally rapid decrease. Very few ‘cough’ absences were reported during summer 2020. During September 2020, a large, rapid increase in ‘cough’ absences was observed, followed by a decrease during October to levels which were similar to 2019. ‘Cough’ absences were lower in November/December 2020 than in 2019.

## Discussion

This study has explored the use of a school absence reporting app to supplement a syndromic surveillance service, focussing here on monitoring COVID-19 activity. The long-term aim of this system is not to report on levels of absenteeism as a measure of school performance but to monitor trends in absence to support our understanding of community morbidity.

As expected, during 2019 the six troughs observed in the number of absence reports corresponded with school holiday dates in England. The increased absence levels both in ‘total’ and for ‘cough’ absences throughout November and December 2019 may reflect normal seasonal patterns of absence, likely due to the winter circulation of respiratory pathogens [13].

The initial trends of ‘total’ and ‘cough’ absences during January/February 2020 were similar to that of 2019. However, the increase in the number of absence reports made in March 2020 reflects the impact of the first wave of COVID-19 in England. The steep decrease from the end of March through to May 2020 was a likely result of the first national lockdown, which began on 24 March 2020 [14], when school closures were enforced across England. The slight increase in ‘total’ absences during June 2020 coincided with the partial reopening of schools for a subset of children across England [15].

Full school reopening was implemented in September 2020 [16], after which the number of absence reports (‘total’ and for ‘cough’) increased above the comparable period of 2019, but not to the levels seen in March 2020. The increase in September 2020 may reflect several factors similar to those that contributed to patterns observed in late 2019: children mixing with others, many for the first time in several months, giving the opportunity for respiratory pathogens (in addition to COVID-19) to circulate. However, in 2020 subsequent heightened parental concern at the beginning of the school term may have resulted in parents being more likely to keep their children at home if symptoms were mild or even non-existent, adding to levels of absence reported. Absences may also have possibly included the ‘worried well’ and/or, as outlined in the guidelines [17], children or those who lived in the same household that were waiting for COVID-19 test results.

Similar observations were made within other syndromic surveillance reported in England at the time. The trend in the number of paediatric emergency departmental attendances rapidly decreased during March 2020 before a steady increase during the summer, followed by a peak in attendances during September 2020 [18]. Furthermore, unanticipated increases in the number of NHS 111 calls for respiratory type conditions were also observed in children in September 2020 [19].

A strength of the use of the Studybugs app data for the development of a syndromic surveillance system is the real-time availability of standardised school absence report data, including the reason(s) for and symptoms related to absence. Using a single reporting app, we demonstrate the advantage of a single platform used across a number of schools. School absence records also have the advantage that in many countries recording school attendance is a legal requirement and therefore absence data, in general, should be a very complete data source. Here, the ability to identify the reason for absence (e.g. cough) allows for more detailed investigation. Studybugs has previously been used to measure the proportion of and reasons for asthma-related school absence in England [9]. Other studies have used school absence data to illustrate the impact of certain conditions such as influenza [20–22].

This new system is intended to complement existing public health surveillance activities in PHE, rather than be run in isolation. Using school absence data for public health surveillance helps to supplement other sources of surveillance data as it can capture information on illness related to milder symptoms in children, where medical assistance is not sought, and therefore not captured by other public health surveillance. This may be the case for COVID-19, and other illnesses such as gastrointestinal and other respiratory infections. The Studybugs data include anonymised identifiers for episodes of illness which may allow further investigation into duration of absence due to specific symptoms.

**Fig. 3.**
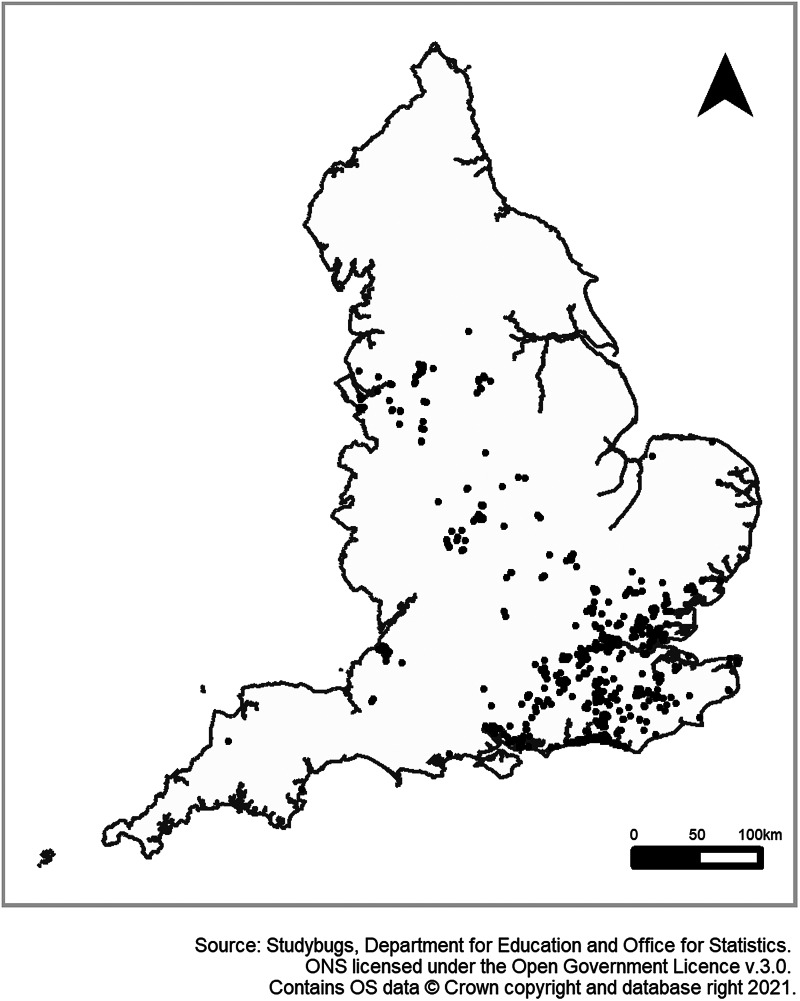
Map of schools participating in Studybugs in 2019 and 2020, England.

This is the first time PHE has used an open dataset from an API data source for syndromic surveillance purposes, rather than working with healthcare advice/providers to access anonymised data collected specifically for health purposes. As with all syndromic surveillance systems, there are some limitations to this new pilot system and the surveillance outputs reported here. Schools using the Studybugs service represent around 3.3% of schools in England and participation can vary. This study included only schools that provided data in both 2019 and 2020. Further to this, the current representation of schools included in the data received is heavily skewed to the South East of England with almost 50% of the data coming from schools in this region (Fig. 3). As a pilot sentinel system, this coverage will be monitored and the messaging for public health purposes will be tailored accordingly. The biggest improvement to this pilot sentinel syndromic surveillance system would be the inclusion of more schools with better coverage of all areas across England, not just the South East region.

Syndromic surveillance by design does not use data that has been validated by medical professionals or laboratory results, instead of using health information collected and made available in near real-time to report trends as quickly as possible. In this new pilot system, the Studybugs data are based on parents’ data input, not verified by trained medical professionals. It is assumed that any potential bias in reporting is consistent throughout the period under investigation allowing for identification of trend.

Another weakness is absence may be submitted for the next day from 6 pm on the previous day, therefore analysis by date of entry into the app (as used here) may not accurately record when the absence occurred. Also, during school holidays and periods of national lockdown (when many schools were closed) school absence surveillance cannot inform on the health of the population under surveillance as the data is likely to be less complete than on days when a child is expected to physically attend school.

Further work is required to investigate the most appropriate way to report trends, possibly as rates (registered school population numbers are available) rather than numbers as we have presented here. Additionally, standardised statistical analyses are in place for syndromic surveillance in England, these are to be developed for application to this new data source, with the consideration required for the effects of school holidays in particular. The future statistical interrogation of the data will be required for this pilot to develop into a live system.

This paper illustrates the use of an open data source for real-time surveillance to monitor school absence during the COVID-19 pandemic. This pilot sentinel syndromic surveillance system shows potential for complementing other methods of COVID-19 surveillance, providing context for national absence levels in schools. As well as looking at school absences overall, one further advantage of the data is that they can be used to illustrate trends by different syndromic conditions including ‘cough’, ‘fever’ and more recently ‘COVID-19’ and ‘loss of smell/taste’. Additionally, the impact of influenza-like illnesses and gastrointestinal infections on school absence could also be investigated using these data, highlighting the potential for Studybugs to become a flexible wide-ranging surveillance tool.

## Data Availability

Data used is freely available at source (https://studybugs.com/).
